# 超高效合相色谱法拆分和测定克伦特罗对映体

**DOI:** 10.3724/SP.J.1123.2021.06045

**Published:** 2021-12-08

**Authors:** Wenhua ZHANG, Deng HONG, Meikang LEI, Xiaoli HU, Jianbo HOU, Wen XIE, Dunming XU, Xionghai YI, You LI

**Affiliations:** 1.杭州海关技术中心, 浙江 杭州 310016; 1. Technic Center of Hangzhou Customs, Hangzhou 310016, China; 2.浙江省检验检疫科学技术研究院, 浙江 杭州 310016; 2. Zhejiang Academy of Science and Technology for Inspection and Quarantine, Hangzhou 310016, China; 3.厦门海关技术中心, 福建 厦门 361026; 3. Technical Center of Xiamen Customs, Xiamen 361026, China; 4.上海海关动植物与食品检验检疫技术中心, 上海 200135; 4. Technical Center for Animal, Plant and Food Inspection and Quarantine, Shanghai Customs, Shanghai 200135, China

**Keywords:** 超高效合相色谱, 克伦特罗, 对映体, 手性分离, ultra-performance convergence chromatography (UPC^2^), clenbuterol, enantiomers, chiral separation

## Abstract

建立了基于超高效合相色谱技术(UPC^2^)的克伦特罗对映体拆分方法,并对所建立的方法进行了方法学考察及应用。实验考察了两种克伦特罗对映体标准溶液的稳定性,并对手性色谱柱、助溶剂、系统背压、色谱柱温度等色谱分离条件进行了优化。采用Acquity Trefoil AMY1 (150 mm×3.0 mm, 2.5 μm)手性色谱柱进行分离,以超临界CO_2_-含0.5%(v/v) 10 mol/L醋酸铵的甲醇溶液为流动相,以流速2.0 mL/min梯度洗脱,检测波长为241 nm,进样体积为10 μL,系统背压为13.8 MPa,柱温为40 ℃时,两种克伦特罗对映体分离效果最好。两种克伦特罗对映体的线性范围为1.0~20.0 mg/L,相关系数均大于0.9997,仪器检出限(*S/N*=3)均为0.5 mg/L。10.0 mg/L混合标准工作溶液重复进样6次,(+)、(-)克伦特罗对映体峰面积的相对标准偏差(RSD, *n*=6)分别为0.65%和0.76%。应用该方法对市售克伦特罗外消旋体标准品进行拆分,采用外标定量法计算克伦特罗外消旋体标准中间溶液10.0 mg/L中两种克伦特罗对映体含量,其中(+)-克伦特罗的含量为5.6 mg/L, (-)-克伦特罗的含量为5.5 mg/L。该计算结果与文献报道的工业品克伦特罗外消旋体中(+)-克伦特罗与(-)-克伦特罗的比例为1.02:1.00基本相符。该方法具有分析速度快、分离效果好、有机溶剂消耗少等特点,适用于克伦特罗对映体的拆分,为其他手性化合物的拆分、药效精细分析和产品质量评定提供了可靠的技术支持。

## Abstract (en)

Clenbuterol enantiomers differ greatly in their bioactivities. By optimizing the conditions for chromatographic separation and method validation, ultra-performance convergence chromatography (UPC^2^) was adopted to separate the enantiomers of clenbuterol. Standard solutions of (+)-clenbuterol and (-)-clenbuterol were stored at -18 ℃ for 1, 3, 5, 7, 14, 30, and 60 d, and then, their stability was monitored. The impacts of different chromatographic columns, cosolvents, system backpressure, and chromatographic column temperature on the separation of the two enantiomers were investigated. Acquity Trefoil AMY1 (150 mm×3.0 mm, 2.5 μm) was used for separation, and CO_2_-0.5% (v/v) ammonium acetate was used as the mobile phase. Gradient elution at a flow rate of 2.0 mL/min was adopted. The detection wavelength was set to 241 nm, and the injection volume was set to 10 μL. The backpressure was set to 13.8 MPa, and the column temperature was maintained at 40 ℃. The two enantiomers showed good linear relationships in the range of 1.0 to 20.0 mg/L with correlation coefficients greater than 0.9997. The limits of detection (LODs, *S/N*=3) of (+)-clenbuterol and (-)-clenbuterol were both 0.5 mg/L. The relative standard deviation (RSD, *n*=6) for the peak area of the 10.0 mg/L mixed standard working solution with six replicate injections ranged from 0.65% to 0.76%. The effectiveness and practicability of this method were demonstrated by using it to detect standard clenbuterol racemate. The (+)-clenbuterol and (-)-clenbuterol contents were 5.6 mg/L and 5.5 mg/L, respectively, in the standard clenbuterol racemates, as determined by the external standard method of quantification. The detection results suggested that the content ratio of (+)-clenbuterol and (-)-clenbuterol was close to 1.02:1.00, which is consistent with the literature data. The established method has the advantages of rapid analysis, good separation effect, and low consumption of organic solvents, and it is suitable for the separation of clenbuterol enantiomers. This method can also provide technical support for the separation of other chiral drugs, analysis of the effects of chiral drugs, and assessment of product quality.

克伦特罗(clenbuterol),化学名为1-(4-氨基-3,5-二氯苯基)-2-(叔丁基-D9-氨基)乙醇,是一种选择性*β*_2_-肾上腺素受体激动剂,临床上主要作为支气管解痉药物,对支气管哮喘和伴有可逆性气道阻塞的慢性支气管炎均有良好效果^[1]^。临床上使用的*β*_2_肾上腺受体类激动剂药物为克伦特罗的外消旋体^[2]^,其分子中含有1个手性碳原子^[3]^,存在一对对映体,包括(+)-克伦特罗对映体和(-)-克伦特罗对映体(见图1)。据文献^[4,5]^报道,不同结构对映体将会产生不同的药理活性。研究发现,克伦特罗外消旋体中不同结构对映体之间的生物活性差异较大,其中(-)-克伦特罗在临床上有疗效,而(+)-克伦特罗则无疗效,故对克伦特罗的手性分离具有重要意义^[6,7]^。为了进一步研究克伦特罗对映体之间的生物活性差异,迫切需要建立一种克伦特罗对映体的高效分离方法。

**图1:**
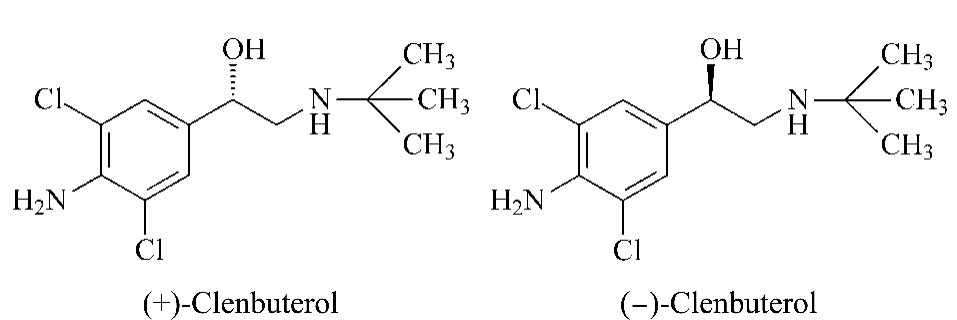
克伦特罗两种对映体的结构式

目前克伦特罗对映体的检测方法主要为高效液相色谱法(HPLC)^[8,9]^、毛细管电泳法^[2,10]^、液相色谱-串联质谱法^[11,12,13,14]^等。其中HPLC分离度好,但有机试剂消耗量大;毛细管电泳法峰形良好,但分析时间长;液相色谱-串联质谱法准确性高,但是仪器昂贵、成本高。近年来超高效合相色谱技术(UPC^2^)受到了广泛关注,该技术以超临界二氧化碳为流动相主体,依靠流动相的溶剂化能力来进行分离、分析,并通过精确调节流动相比例、色谱柱温度和系统背压来精准调控待测物手性化合物的保留时间和分离度^[15]^。超临界流体为流动相使得UPC^2^的分离分析能力克服了气相色谱法(GC)和HPLC的不足之处,既能分析不适用于GC的高沸点、低挥发、遇热不稳定的样品,又能提高HPLC的分析速度和柱效。研究表明,UPC^2^技术更适合于分析传统液相色谱难以处理的结构类似物和同分异构体,已被成功应用于三唑类农药^[16]^、酚酸类化合物^[17]^、色素^[18]^、酚类精油^[19]^等化合物的拆分和测定。目前,UPC^2^技术应用于克伦特罗对映体的拆分及含量测定未见有报道。

本研究建立了超高效合相色谱法拆分和测定克伦特罗对映体的方法。实验考察了两种克伦特罗对映体标准品的稳定性,优化了克伦特罗对映体的色谱分离条件,对所采购的克伦特罗外消旋体标准品进行了拆分及测定。

## 1 实验部分

### 1.1 仪器、材料与试剂

Acquity超高效合相色谱仪(美国Waters公司,带有二极管阵列(PDA)检测器); AE260电子天平(瑞士Mettler公司); R215旋转蒸发仪(瑞士Buchi公司); ELGA CLXXXUVM2超纯水净化系统(英国Elga公司); MS2涡旋混匀器(上海医大仪器厂); N-EVAP^TM^ 111氮吹仪(日本东京理化公司)。

乙腈、甲醇、甲酸、乙醇、异丙醇、正庚烷(色谱纯,西班牙Scharlau公司);醋酸铵、氨水(优级纯);超纯水;高纯二氧化碳(99.999%);其他实验所用试剂除特殊说明外均为分析纯。

克伦特罗外消旋体标准品(CAS号:129138-58-5,纯度≥98.0%, BePure公司)。两种克伦特罗对映体标准品:(+)-克伦特罗、(-)-克伦特罗由上海勤路生物技术有限公司从克伦特罗外消旋体标准品(BePure公司)中分离纯化获得,纯度均大于98.0%。

### 1.2 标准储备液及工作液的配制

1.2.1 外消旋体标准储备液

准确称取0.01 g(精确至0.1 mg)克伦特罗外消旋体标准品,用甲醇溶解并定容至10 mL,配成1.0 g/L的外消旋体标准储备液。

克伦特罗外消旋体的标准中间溶液:准确吸取一定量的外消旋体标准储备液,用乙腈稀释至10.0 mg/L的标准中间溶液。

1.2.2 对映体标准储备液

分别准确称取0.01 g(精确至0.1 mg)(+)-克伦特罗和(-)-克伦特罗标准品,用甲醇溶解并定容至10 mL,配成1.0 g/L的对映体标准储备液。

两种克伦特罗对映体的混合标准工作溶液:分别准确吸取一定量的(+)-克伦特罗和(-)-克伦特罗对映体标准储备液,用乙腈逐级稀释至1.0、2.0、4.0、10.0、20.0 mg/L的混合标准工作溶液。

### 1.3 分析条件

色谱柱:Acquity Trefoil AMY1 (150 mm×3.0 mm, 2.5 μm);流动相:A为CO_2_, B为含0.5%(v/v) 10 mol/L醋酸铵的甲醇溶液;梯度洗脱程序:0~2.0 min, 7%B; 2.0~2.1 min, 7%B~18%B; 2.1~4.25 min, 18%B; 4.25~4.3 min, 18%B~7%B; 4.3~6.3 min, 7%B。系统背压:13.8 MPa;流速:2.0 mL/min;进样量:10 μL;柱温:40 ℃;检测波长:241 nm。

## 2 结果与讨论

### 2.1 检测波长的选择

通过PDA检测器扫描后,从色谱图上提取克伦特罗对映体标准溶液的紫外光谱图。如图2所示,在209、241、296 nm处均有明显的吸收峰,其中209 nm处的吸收最强,灵敏度相对较高,但在该波长下克伦特罗对映体出峰处杂质干扰峰较多;241 nm处吸收较强,克伦特罗对映体出峰处干扰峰较少。综合考虑,对克伦特罗药品检测而言,用吸光度较高且杂质较少的241 nm波长检测更具有优势,故本实验选择241 nm作为检测波长。

**图2:**
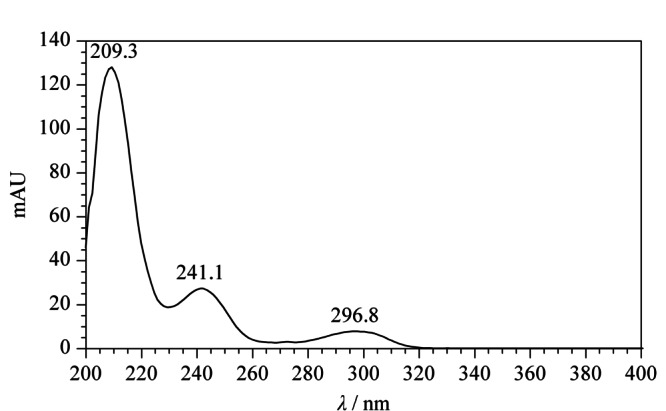
克仑特罗对映体标准溶液的光谱图

### 2.2 色谱柱的优化

基于直链淀粉-三(3,5-二甲基苯基氨基甲酸酯)与纤维素-三(3,5-二甲基苯基氨基甲酸酯)的手性固定相是应用最为广泛的两类固定相,具有良好的手性识别能力和拆分能力,在手性识别能力方面互为补充^[20]^。本实验选择美国Waters公司的Acquity Trefoil CEL2 (150 mm×3.0 mm, 2.5 μm,填料为纤维素-三(3-氯-4-甲基苯基氨基甲酸酯))、Acquity Trefoil AMY1(150 mm×3.0 mm, 2.5 μm,填料为直链淀粉-三(3,5-二甲基苯基氨基甲酸酯))、Acquity Trefoil CEL1(150 mm×3.0 mm, 2.5 μm,纤维素-三(3,5-二甲基苯基氨基甲酸酯))和大赛璐药物手性技术(上海)有限公司的多糖衍生物耐溶剂型手性色谱柱CHIRALPAK IA-3(100 mm×4.6 mm, 3 μm,硅胶表面共价键合有直链淀粉-三(3,5-二甲基苯基氨基甲酸酯))、纤维素衍生物正相手性柱CHIRALPAK OJ-H(100 mm×4.6 mm, 5 μm,表面涂敷了手性多聚物(直链淀粉或纤维衍生物)的球形硅胶)共5种手性分离色谱柱对两种克伦特罗对映体的拆分效果进行考察。

结果表明,采用OJ-H和CEL1手性色谱柱分离时,两种克伦特罗对映体未能实现完全分离;采用CEL2和IA-3手性色谱柱分离时,分离度良好,但色谱峰形展宽明显;而采用AMY1手性色谱柱分离时,分离度良好,且色谱峰形尖锐(见图3),其拆分机理为克伦特罗对映体的官能团与固定相上的手性空腔相互作用,通过“三点作用”方式^[21]^进行手性识别,两个对映体与固定相的作用力有差异,使得保留时间不同。AMY1手性固定相分子中的位阻基团与(+)-克伦特罗对映体分子中的直链氨基烷基之间形成了强烈的位阻作用,导致两者手性识别所需的分子间作用力被阻碍,其在手性固定相上的有效吸附也相应被减弱,故(+)-克伦特罗对映体分子被流动相优先洗脱而先出峰;而(-)-克伦特罗对映体分子具有适宜的空间构型,如图1结构式所示,羟基无明显的位阻效应,能容易与固定相的酯基形成氢键作用力,有利于产生“三点作用”,保留时间延长,因而可被拆分。OJ-H、CEL1和AMY1的固定相填料不同,在超临界CO_2_-0.5%(v/v) 10 mol/L醋酸铵甲醇溶液流动相体系中,两个克伦特罗对映体在OJ-H和CEL1柱上未达到基线分离;AMY1和IA-3的固定相相同,但AMY1固定相的内径和填料粒径比IA-3小,故色谱峰形更尖锐。因此本实验选择AMY1手性色谱柱对克伦特罗对映体进行分离。

**图3:**
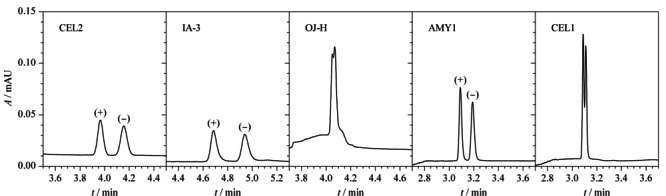
不同色谱柱对(+)-克伦特罗和(-)-克伦特罗分离效果的影响

### 2.3 流动相中助溶剂的选择

超高效合相色谱的有机溶剂消耗量少,采用超临界CO_2_为主要流动相,通常使用少量有机溶剂作为助溶剂,以加强对目标产物的洗脱能力和选择性。本实验考察了0.5%(v/v)甲酸甲醇溶液、0.5%(v/v) 10 mol/L醋酸铵甲醇溶液、0.5%(v/v)氨水甲醇溶液等不同助溶剂对两种克伦特罗对映体分离的影响。结果显示,当使用0.5%(v/v)甲酸甲醇溶液作为助溶剂时,两种克伦特罗对映体未能出峰;当使用0.5%(v/v) 10 mol/L醋酸铵甲醇溶液和0.5%(v/v)氨水甲醇溶液作为助溶剂时,两种克伦特罗对映体的色谱峰均在4.0 min内实现了完全分离,但0.5%(v/v)氨水甲醇溶液助溶剂时,两个对映体色谱峰的信噪比(*S/N*)分别为28和20, 0.5%(v/v) 10 mol/L醋酸铵甲醇溶液作为助溶剂时,两个对映体色谱峰的信噪比(*S/N*)分别为35和26(见图4)。因此,本实验室选择0.5%(v/v) 10 mol/L醋酸铵甲醇溶液作为助溶剂。

**图4:**
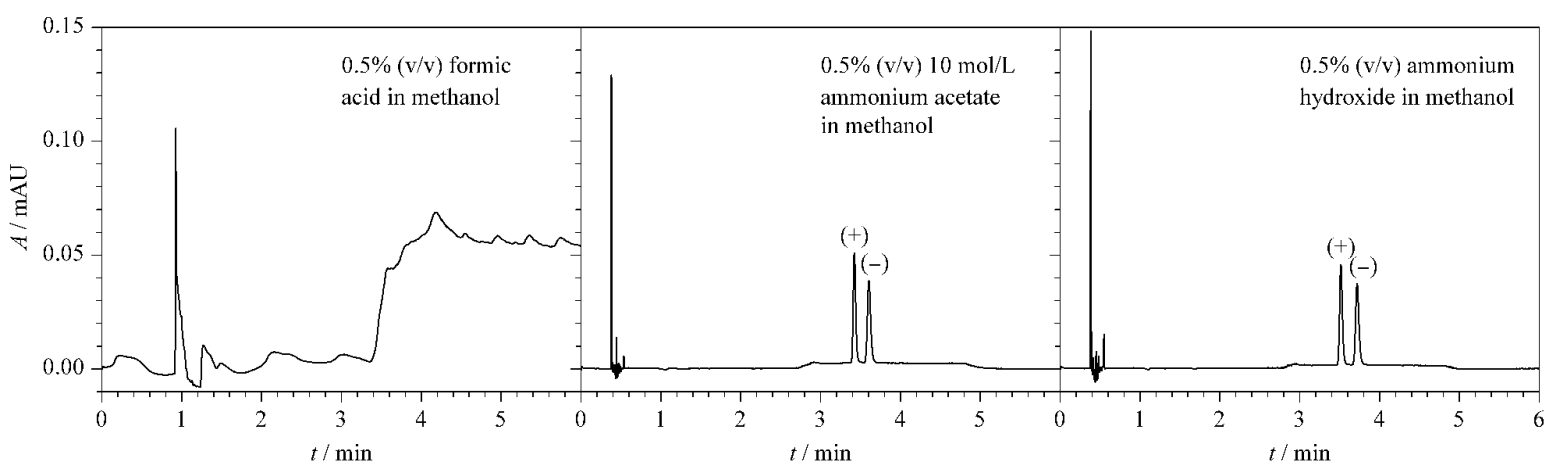
不同助溶剂对(+)-克伦特罗和(-)-克伦特罗分离效果的影响

### 2.4 系统背压的选择

UPC^2^采用超临界状态CO_2_作为流动相,通过调整系统背压和温度可有效改变CO_2_的密度,从而改变其对物质的溶解能力、洗脱能力和选择性。由于CO_2_的温度超过31 ℃且压力超过7.38 MPa以上,CO_2_才会进入超临界状态。因此本实验以0.5%(v/v) 10 mol/L醋酸铵甲醇溶液作为助溶剂,在柱温40 ℃条件下考察系统背压在10.3~20.7 MPa范围内对两种克伦特罗对映体分离的影响。结果显示,随着系统背压的升高,分析物的保留时间提前(见图5)。4种条件下,两种克伦特罗对映体的分离度分别为1.7、1.8、1.6、1.5,在系统背压为13.8 MPa时的色谱峰分离度达到最佳,故本研究选择背压为13.8 MPa。

**图5:**
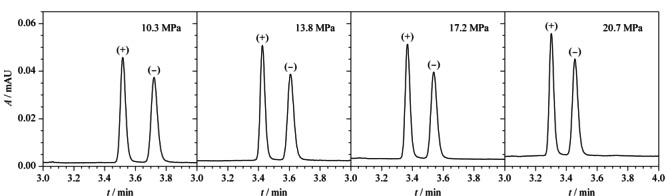
不同系统背压对(+)-克伦特罗和(-)-克伦特罗对映体分离效果的影响

### 2.5 色谱柱温度的选择

在UPC^2^系统中,色谱柱温度主要通过影响流动相密度,从而影响对目标物的分离效果。随着色谱柱温度升高,CO_2_超临界流体的黏度降低,密度减小,对目标产物的洗脱能力也随之减小,保留时间延长。考虑到Acquity Trefoil AMY1手性色谱柱的最高推荐运行温度为40 ℃, CO_2_的温度超过31 ℃且压力超过7.38 MPa以上,CO_2_才会进入超临界状态。因此本实验考察了色谱柱温在31~40 ℃范围内对克伦特罗对映体分离的影响。结果表明,随着柱温升高,目标物的保留时间逐渐延长(见图6)。3种柱温条件下,两种克伦特罗对映体的分离度分别为1.3、1.5、1.7,在柱温为40 ℃时的色谱峰分离度达到最佳,4.0 min内实现良好的基线分离,分析速度快。因此,选择最佳色谱柱温度为40 ℃。

**图6:**
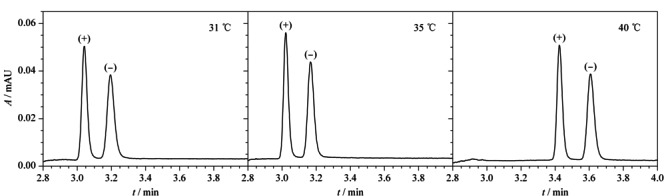
不同色谱柱温度对(+)-克伦特罗和(-)-克伦特罗分离效果的影响

### 2.6 定容试剂的选择

使用5种定容试剂:甲醇、乙醇、乙腈、异丙醇、正庚烷对10 mg/L的克伦特罗对映体进行拆分,结果如图7所示,当用甲醇作为定容试剂时,目标峰中包含杂质峰;当用乙醇作为定容试剂时,目标物峰形较差;当用乙腈、异丙醇和正庚烷作为定容试剂时,两种克伦特罗对映体的色谱峰均在4.0 min内实现了完全分离,但相比异丙醇和正庚烷两种定容试剂,乙腈作为定容试剂时,目标物的色谱峰分离度更好,峰形更尖锐。因此,最后确定乙腈为本实验定容试剂。

**图7:**
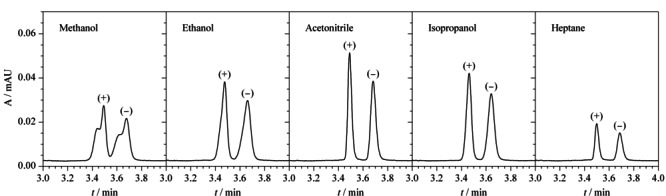
不同定溶试剂对(+)-克伦特罗和(-)-克伦特罗分离效果的影响

### 2.7 方法学考察

2.7.1 线性范围和灵敏度

将(+)-克伦特罗和(-)-克伦特罗的系列混合标准溶液按上述色谱条件进行测定。以标准品的峰面积(*Y*)为纵坐标,对应质量浓度(*X*)为横坐标,绘制标准曲线,求得回归方程和相关系数。结果表明,两种克伦特罗对映体在1.0~20.0 mg/L质量浓度范围内呈良好的线性关系,相关系数大于0.9997。当信噪比为3(*S/N*=3)时,两种克伦特罗对映体的仪器检出限(LOD)分析结果见表1。

**表1:** 克伦特罗对映体的线性范围、线性方程、相关系数和检出限

Clenbuterol enantiomer	Linear range/(mg/L)	Linear equation	r^2^	LOD/(mg/L)
(+)-Clenbuterol	1.0-20.0	Y=1.48×10^4^X+4.34×10^3^	0.9997	0.5
(-)-Clenbuterol	1.0-20.0	Y=1.30×10^4^X+5.26×10^2^	0.9998	0.5

*Y*: peak area; *X*: mass concentration, mg/L.

2.7.2 精密度

取10.0 mg/L混合标准工作溶液,重复进样6次,按1.3节色谱条件进行测定,计算结果表明(+)、(-)-克伦特罗对映体峰面积的相对标准偏差(RSD, *n*=6)分别为0.65%和0.76%,该测定方法的精密度符合GB/T 32465-2015^[22]^的要求,能够满足克伦特罗对映体的拆分和测定要求。

### 2.8 克伦特罗对映体标准溶液稳定性的考察

分别准确移取1.0 mL的10 mg/L克伦特罗对映体的混合标准工作溶液于7个带划痕的1.5 mL UPC^2^专用进样小瓶中,上机进行测定,检测后再转移至7个带铝盖密封的进样小瓶中,并用封口膜封好后于-18 ℃下保存放置。将新配制的质量浓度为10.0 mg/L的两种克伦特罗对映体标准溶液,与分别储存1、3、5、7、14、30、60天后的10.0 mg/L克伦特罗对映体的测定结果作图比较,以新配制标准溶液作为100%,克伦特罗对映体标准溶液变化小于10%作为基准。结果显示,两种克伦特罗对映体测定结果都是呈逐渐降低趋势(见图8),其中两种克伦特罗对映体于-18 ℃下放置60天时含量降低了20%以上,放置30天时含量变化小于10%,而放置7天时含量变化小于5%,表明两种克伦特罗对映体在30天内比较稳定。

**图8:**
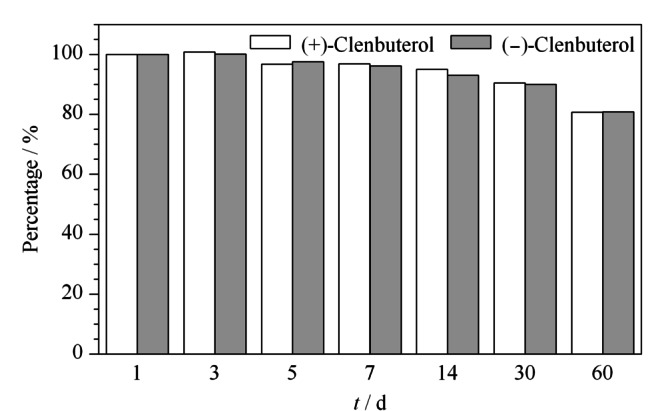
(+)-克伦特罗和(-)-克伦特罗标准溶液60天内的稳定性考察(乙腈溶液)

### 2.9 方法的应用

采用本文建立的方法对所采购的克伦特罗外消旋体标准品进行拆分及测定。如图9所示,两种克伦特罗对映体的分离效果良好,在4.0 min内实现了有效拆分,分离度为1.7,符合*R*≥1.5完全分离的要求^[23]^。按照色谱峰的保留时间顺序,依次为(+)-克伦特罗、(-)-克伦特罗。根据上述所绘制的标准曲线,采用外标定量法计算1.2.1节中克伦特罗外消旋体标准中间溶液10.0 mg/L的两种克伦特罗对映体含量,其中(+)-克伦特罗的含量为5.6 mg/L, (-)-克伦特罗的含量为5.5 mg/L。计算结果与文献^[12]^报道的工业品克伦特罗外消旋体中(+)-克伦特罗与(-)-克伦特罗的比例为1.02∶1.00基本相符。

**图9:**
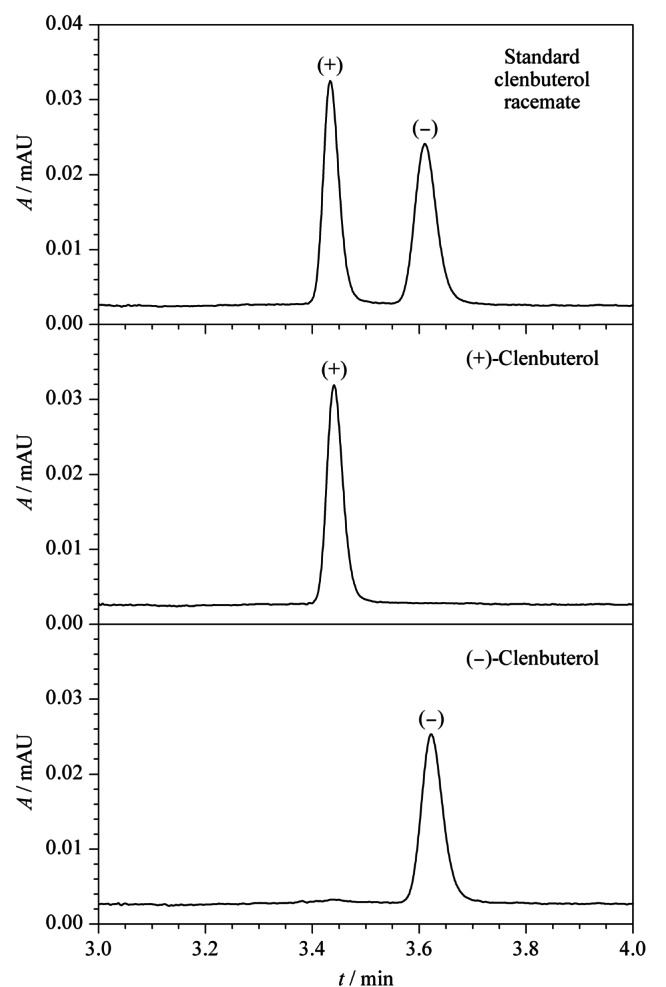
克伦特罗外消旋体的拆分

## 3 结论

本文采用超高效合相色谱技术对克伦特罗对映体进行分离,考察了手性色谱柱、助溶剂、系统背压、柱温、定容试剂对克伦特罗对映体分离的影响,最终确定分离条件为:手性色谱柱为Acquity Trefoil AMY1 (150 mm×3.0 mm, 2.5 μm),助溶剂为0.5%(v/v) 10 mol/L醋酸铵甲醇溶液,流速为2.0 mL/min,检测波长241 nm,柱温40 ℃,系统背压为13.8 MPa。最佳实验条件下的运行时间仅为4.0 min,能够实现对克伦特罗对映体的基线分离,为其他手性化合物的拆分、药效精细分析和产品质量评定提供了可靠的技术支持。

## References

[1] Wu J S. Pharmacology. Beijing: People’s Sanitary Publishing Press, 1998

[2] Huang K L, Hu W G, Wang W L, et al. Journal of Analytical Science, 2007,23(1):9

[3] Thevis M, Thomas A, Beuck S, et al. Rapid Commun Mass SP, 2013,27(4):507 10.1002/rcm.6485 23322656

[4] Gausepopl G J, Blaschke B. Biomed Sci Appl, 1998,713(2):443 10.1016/s0378-4347(98)00178-9 9746262

[5] Abou-Basha L I, Aboul-Enein H Y. Biomed Chromatogr, 1996,10(2):69 8924729 10.1002/(SICI)1099-0801(199603)10:2<69::AID-BMC554>3.0.CO;2-H

[6] Waldeck B, Windmark E. Acta Pharmacol Toxicol, 1985,56:221 10.1111/j.1600-0773.1985.tb01279.x 2990158

[7] Martin P, Puech A J P, Brochet D, et al. Eur J Pharmacol, 1985,117:127 2867910 10.1016/0014-2999(85)90481-9

[8] Wu Y L, Yang T, Shan J H, et al. Chinese Journal of Analytical Chemistry, 2010,38(6):833

[9] Tang Q, Song H, Fu C, et al. Chinese Journal of Analytical Chemistry, 2004,32(6):755

[10] Lv L L, Wang L J, Li J, et al. J Pharmaceut Biomed, 2017,145:399 10.1016/j.jpba.2017.06.044 28719814

[11] Chen G, Liu Y J, Lü Y, et al. Meat Research, 2015,29(5):22

[12] Wang Z L, Zhang J L, Zhao Y, et al. Chinese Journal of Sports Medicine, 2015,34(12):1186

[13] Benjamín V B, Jahir B, Martha E R, et al. J Anal Toxicol, 2020,44(3):237 31681961

[14] Liu Y J, Xu W, Zhang H, et al. Electrophoresis 2019,40(21):2828 31238386 10.1002/elps.201900149

[15] Ultra-Performance Convergence Chromatography(UPC^2^): New Categories of Chromatography Have Empowered Scientists With New Imaginations. (2012-05-15) [2021-06-27]. https://www.antpedia.com/index.php?action-viewnews-itemid-212253-php-1https://www.antpedia.com/index.php?action-viewnews-itemid-212253-php-1

[16] Zhang W H, Xie W, Hou J B, et al. Chinese Journal of Chromatography, 2019,37(12):1356 34213139 10.3724/SP.J.1123.2019.08028

[17] Jiang H, Yang L, Xing X D, et al. J Pharmaceut Biomed Anal, 2018,153:117 10.1016/j.jpba.2018.02.027 29477129

[18] Yu W S, Liu X, Zhang Y Z, et al. Anal Lett, 2020,53(10):1654

[19] Chang X Q, Sun P, Ma Y, et al. Molecules, 2020,25(3):502 10.3390/molecules25030502 PMC7037148 31979387

[20] Yashima E, Okamoto Y. Angew Chem Int Ed, 1998,37(8):1020 10.1002/(SICI)1521-3773(19980504)37:8<1020::AID-ANIE1020>3.0.CO;2-5 29711008

[21] William H P, Thomas C P. J Chem Rev, 1989,89:347

[22] GB/T 32465-2015

[23] Separation Degree. [2021-07-10]. https://baike.baidu.com/item/%E5%88%86%E7%A6%BB%E5%BA%A6/11007800?fr=Aladdinhttps://baike.baidu.com/item/%E5%88%86%E7%A6%BB%E5%BA%A6/11007800?fr=Aladdin

